# Metronomic oral vinorelbine in patients with advanced non-small cell lung cancer progressing after nivolumab immunotherapy: a retrospective analysis

**DOI:** 10.3332/ecancer.2020.1113

**Published:** 2020-09-29

**Authors:** Vittorio Gebbia, Marco Maria Aiello, Giuseppe Banna, Giusi Blanco, Livio Blasi, Nicolò Borsellino, Dario Giuffrida, Mario Lo Mauro, Gianfranco Mancuso, Dario Piazza, Giuseppina Savio, Hector Soto Parra, Maria Rosaria Valerio, Francesco Verderame, Paolo Vigneri

**Affiliations:** 1 Medical Oncology Unit, La Maddalena Clinic for Cancer Medical Oncology, Palermo 90100, Italy; 2 PROMISE Department, University of Palermo, Palermo 90100, Italy; 3 Policlinico-Vittorio Emanuele, Università di Catania, Catania 95100, Italy; 4 Medical Oncology Unit, Ospedale Cannizzaro, Catania 95100, Italy; 5 Medical Oncology Unit, IOM, Catania 95100, Italy; 6 Medical Oncology Unit, ARNAS Civico, Palermo 90100, Italy; 7 Medical Oncology Unit, Ospedale Buccheri La Ferla, Palermo 90100, Italy; 8 Fondazione GSTU, Palermo 90100, Italy; 9 Medical Oncology Unit, AOUP P. Giaccone, Palermo 90100, Italy; 10 Medical Oncology Unit, Ospedale Cervello/Villa Sofia, Palermo 90100, Italy

**Keywords:** oral vinorelbine, metronomic therapy, non-small cell lung cancer, nivolumab

## Abstract

**Purpose:**

The availability of immune checkpoint inhibitors has deeply changed the therapeutic scenario of patients with advanced non-small cell lung cancer (NSCLC). Up until now, chemotherapy still represents the first-line treatment for patients with advanced NSCLC not harbouring genetic mutations or lacking high expression of programmed death ligand even if the addition of immunotherapy to first-line chemotherapy has recently been shown to improve clinical outcome. We carried out a multi-institutional retrospective analysis on third-line chemotherapy with metronomic oral vinorelbine (VNR) in a series of patients with metastatic NSCLC pre-treated with first-line chemotherapy and second-line immunotherapy.

**Patients and methods:**

Thirty patients with metastatic NSCLC with progressive disease after first-line chemotherapy and subsequent immunotherapy were treated with metronomic oral VNR continuously at the fixed dose of 30 mg three times per week.

**Results:**

A partial response was achieved in 4 patients (13.3%), while 10 patients (33.3%) displayed disease stabilisation for an overall disease control rate of 46.7%. Median progression-free survival was 3.9 months (range 1–13 months) and median OS reached 8.1 months (range 4.0–24.0+ months) with a 12-month survival rate of 22%.

**Conclusion:**

Oral metronomic VNR appears to be active and safe in patients with metastatic NSCLC in progression after first-line chemotherapy and second-line immunotherapy. The results reported, although from a limited sample, may suggest its use for long-term stabilisation of the disease with good patient compliance.

## Introduction

The availability of immune checkpoints inhibitors, such as nivolumab and pembrolizumab, has deeply changed the therapeutic scenario for patients with advanced non-small cell lung cancer (NSCLC) [1]. Up until now, platinum-based chemotherapy doublets still represent the first-line treatment of patients with advanced NSCLC not harbouring genetic alterations of oncogenic drivers or lacking high expression of the programmed death ligand even if the addition of immunotherapy to fist-line chemotherapy has recently been shown to improve clinical results [2, 3]. Fit patients progressing after immunotherapy are often treated with subsequent lines of chemotherapy in daily clinical practice, but reliable scientific data in this disease setting are limited. Preliminary evidence has suggested that the immunotherapy pre-treatment improves response to subsequent chemotherapy in various oncological settings [4]. Indeed, a high objective response rate to third-line docetaxel (29%) has been recently reported in a retrospective study in patients with advanced NSCLC pretreated with platinum-based chemotherapy and immunotherapy as first- and second-line treatments, respectively, suggesting a priming effect of previous immunotherapy [5].

Among chemotherapeutic agents employed in advanced NSCLC vinorelbine (VNR) has shown good anti-tumour activity and acceptable safety both after intravenous and oral administration [6, 7]. In the last decade, oral VNR has also been tested as single agent or in combination with platinum compounds in some phase I-II trials on a metronomic schedule, i.e., the administration of prolonged, non-stop low doses of otherwise active drugs [8–11]. In these studies, a flat dose in the range of 30–50 mg three times per week has been identified as the optimal schedule in terms of tolerability. The metronomic approach is considered intriguing due to its anti-angiogenic effect and the possible synergy with molecularly-targeted agents and immune checkpoints inhibitors [12–14]. Moreover, lack of major side-effects while preserving anti-neoplastic activity makes this schedule highly attractive for the management of heavily pretreated patients or elderly/frail ones [15–17].

In this paper, we report a multi-institutional study on third-line chemotherapy with oral metronomic VNR in a series of patients with metastatic NSCLC pretreated with firs-line chemotherapy and second-line immunotherapy.

## Material and methods

### Study population

Patients included in this analysis had to fulfil the following eligibility criteria: histologically confirmed diagnosis of metastatic NSCLC according to the TNM classification version 7.0 [18]; performance status on the Eastern Cooperative Oncology Group (ECOG) scale of 0–2; measurable disease according to the RECIST criteria [19]; progressive disease after second-line therapy with immune checkpoints inhibitors; adequate renal and hepatic functions; computed tomography scan and or CNS magnetic resonance imaging available for radiological review.

### Treatment schedule

Oral VNR was given continuously at the fixed dose of 30 mg three times per week (Monday, Wednesday and Friday) 30 minutes after a meal. Antiemetic therapy with oral ondansetron was given 1 hour before VNR. Four weeks of treatment were considered as one treatment cycle. The therapy was continued until progression, patient refusal or unacceptable toxicity. Complete blood cell counts and serum chemistry were obtained every 2 weeks.

### Design and statistics

The aim of the study was to assess the activity and toxicity of metronomic oral VNR in a series of unselected patients with metastatic NCSLC after immunotherapy failure in patients progressing after first-line chemotherapy. The size of the statistical sample was not preprogrammed due to the descriptive nature of the analysis. After communication to the Ethical Committee, all clinical information for each eligible individual patient were retrospectively collected employing an anonymous electronic case report form with data directly tabulated on a central server. Objective responses according to the RECIST criteria were reported as absolute numbers and their relative rates; the sum of partial responses and disease stabilisation lasting more than 12 weeks was considered as disease control rate. Progression-free survival (PFS) and overall survival (OS) curves were calculated employing the Kaplan–Meier method. PFS was defined as the time from the beginning of metronomic oral VNR until objective neoplastic progression or death, whichever occurred first. Overall survival was defined as the time interval between the start of treatment and death or last follow-up contact. Side effects were recorded according to the Common Terminology Criteria for Adverse Events v 4.3, which is routinely used at all participating institutions, and was carried out by each investigator in their own institution.

## Results

### Patient population

Databases containing data from 249 advanced NSCLC patients treated in the last 4 years with second-line immune checkpoint inhibitors were queried for the use of metronomic oral VNR. Overall, 30 patients with metastatic NSCLC progressing after first-line chemotherapy and subsequent immunotherapy received oral VNR on a metronomic schedule in eight medical oncology units in Sicily. Table 1 describes the main clinical and demographic characteristics of subjects included in this analysis. Briefly, there were 23 males (77%) and 7 females (23%) with a median age of 69 years (range 49–80) and a median ECOG performance status of 1. Seventy percent of patients had an adenocarcinoma, while 27% were diagnosed with squamous cell carcinoma. Most patients (94%) received first-line chemotherapy with platinum-based doublets, while two individuals had single-agent gemcitabine. All patients received immunotherapy with nivolumab (97%) or pembrolizumab (3%).

### Clinical outcomes

**Figure 1.**
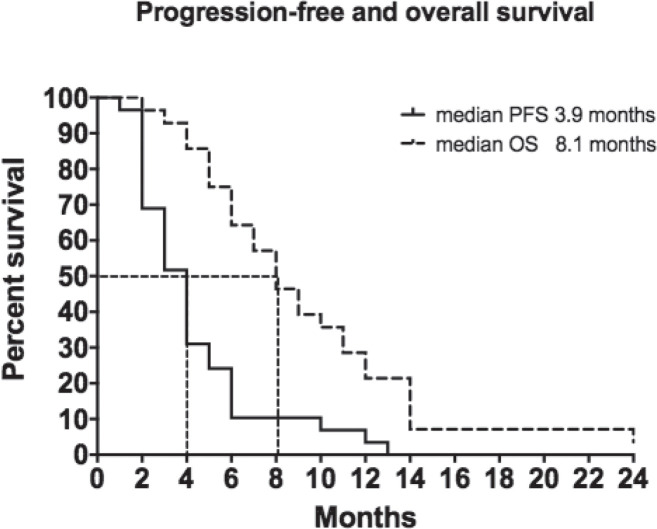
Progression free and overall survival.

As shown in Table 2, 4 patients (13.3%) achieved a partial response and 10 (33.3%) attained disease stabilisation with an overall disease control rate of 46.7%. Sixteen patients (53.3%) progressed. Median PFS was 3.9 months (range 1–13 months) and median OS reached 8.1 months (range 4.0–24.0+ months) with a 22% 12-month survival rate (Figure 1). We found no correlation between objective response rates to VNR and any specific clinical feature. Two thirds of the individuals included in this study received a subsequent line of treatment.

### Safety

Metronomic oral VNR was well tolerated. Overall, 116 cycles of chemotherapy were delivered. Grade 3 toxicities were rare with two patients developing anaemia that required blood transfusion, and one patient with transitory grade 3 neutropenia. Grades 1–2 fatigue and diarrhoea were reported in 8 (27%) and 7 (23%) cases, respectively. Grade 1 neutropenia was reported in five patients (175) but required no further treatment. Four patients (13%) complained of grade 1 constipation. Oral VNR was delayed by at least 1 week in 9 patients (30%).

## Discussion

In the last decade, the administration of oral chemotherapy on a metronomic schedule has raised considerable interest among medical oncologists due to its preclinical rationale and clinical activity and safety [20]. Based on these data and on the observed efficacy of third-line chemotherapy after immunotherapy as compared to historical data [5], we carried out an analysis on the activity and tolerability of third-line chemotherapy with metronomic oral VNR in in patients with metastatic NSCLC pretreated with first-line chemotherapy and second-line immunotherapy.

We report a partial response in 13.3% of patients with disease stabilisation in 33.3% of our cohort with an overall disease control rate of 46.7%. Median PFS was 3.9 months (range 1–13 months) and median OS reached 8.1 months (range 4.0–24.0+ months) with a 12-month survival rate of 22% (Figure 1). These findings are not surprising as other studies failed to identify correlations between patient outcomes and metronomic oral VNR pharmacokinetics [21, 22].

Oral metronomic VNR at the dose of 50 mg/day three times/week has been employed as first-line therapy in elderly and/or unfit patients with interesting results. Camerini *et al* [23] reported a series of 43 patients achieving a 18.6% overall response rate and a disease control rate of 58.1% with a median time to progression of 5 months (range 2–21) and median overall survival of 9 (range 3–29) months with limited grade 3 side-effects. Similar data were also reported in another series of 66 patients treated with the same VNR schedule [24]. Banna *et al* [15] employed fist-line metronomic oral VNR at the dose of 30 mg/day in a series of 50 elderly/unfit patients with an overall disease control rate of 32%, 44% and 26% in first and subsequent lines, respectively. Median OS and PFS were 7.3 months and 2.7 months, respectively.

The activity and safety of metronomic oral VNR in second and subsequent lines has been described in three studies. The Hellenic Oncology Research Group reported a series of 26 patients with advanced NCSLC achieving an 11% overall response rate, a median PFS of 2.2 months and a median overall survival of 9.4 months with a 1-year survival rate of 30% [25]. Major side-effects were represented by grade 3 fatigue (11%), neutropenia (8%–24%) and febrile neutropenia in 11%. A Chinese study reported an 8% partial response rate and 42% stable disease rate for a disease control rate of 50% in a series of 26 patients [26]. The median number of treatment cycles received was 2 (range 1–8), while median PFS was 2 months with a median follow-up time of 4 months (range 2–12). Good performance status (ECOG 1) was associated with longer PFS as compared to patients with ECOG 2 performance status, while no correlation was observed with sex, age, smoking status and histology. Major toxicity was grade 3 neutropenia in less than 10% of cases.

Finally, a multi-centre international retrospective real world analysis reported a series of 270 patients with advanced NCSLC with important co-morbid disease treated with oral metronomic VNR as first-, second- or third-line therapy [27]. The 38 patients treated with third-line oral metronomic VNR achieved a TTP of 4.0 months (range 1–19) months and a median OS of 6.5 months (range 2–29). The treatment was well tolerated with grade 3 neutropenia and fatigue observed in 2% of cycles. A significant percentage of patients achieved prolonged disease control and therefore continued their oral VNR. Moreover, patients treated with subsequent immunotherapy after first-line metronomic oral VNR achieved an OS that was 14 months longer than that of the entire recruited population. Authors speculated on the possible association of these results with the anti-angiogenic and/or immune stimulating properties of the metronomic approach.

## Conclusion

Our findings in an unselected patient population, although retrospective and in a small sample, suggest that oral metronomic VNR is an active and well-tolerated treatment for patients with metastatic NSCLC progressing after second-line immunotherapy. In our opinion, the good disease control rate achieved as a third-line treatment and good tolerability suggest that this pharmacological approach is a reasonable therapeutic option in this clinical setting.

## Conflicts of interest disclosure

The author reports no conflicts of interest in this work.

## Authors’ contributions

Study concepts and design: VG; data acquisition and analysis: VG, DP; manuscript preparation, editing, and review: all authors.

## Funding

Not applicable.

## Figures and Tables

**Table 1.** Patient clinical and demographic characteristics.

		Number of patients	Percent
		30	100
Age (years)	Median	69	
Sex	Male	23	77
Sex	Female	7	23
Smoke	Never smoker	8	27
	Former smoker	22	73
Histology	Adenocarcinoma	21	70
	Squamous cell	8	27
	Large cell	1	3
EGFR status	Wild type	26	87
	Mutant	4	13
ALK	Wild-type	30	100
Performance status	ECOG 0	4	13
	ECOG 1	24	80
	ECOG 2	2	7
Disease sites	Lung	27	90
	Nodes	9	30
	Brain	8	27
	Bone	9	30
	Pleura	2	6
	Liver	4	13
	Peritoneum	1	3
Previous treatments	First-line chemotherapy	30	100
	Cisplatin-based	28	94
	Single-agent	2	6
	Second-line therapy	30	100
	Nivolumab	29	97
	Pembrolizumab	1	3

**Table 2.** Clinical outcomes.

	N°	%
Enrolled patients	30	100
Partial response	4	13.3
Stable disease	10	33.3
CBR	14	46.7
Progressive disease	16	53.3
